# Melt-Mixed PP/MWCNT Composites: Influence of CNT Incorporation Strategy and Matrix Viscosity on Filler Dispersion and Electrical Resistivity

**DOI:** 10.3390/polym11020189

**Published:** 2019-01-22

**Authors:** Petra Pötschke, Fanny Mothes, Beate Krause, Brigitte Voit

**Affiliations:** 1 Leibniz-Institut für Polymerforschung Dresden e.V., Hohe Str. 6, 01069 Dresden, Germany; krause-beate@ipfdd.de (B.K.); voit@ipfdd.de (B.V.); 2 Technische Universität Dresden, 01062 Dresden, Germany

**Keywords:** carbon nanotubes, polypropylene, melt mixing, dispersion, electrical properties

## Abstract

Small-scale melt mixing was performed for composites based on polypropylene (PP) and 0.5–7.5 wt % multiwalled carbon nanotubes (MWCNT) to determine if masterbatch (MB) dilution is a more effective form of nanofiller dispersion than direct nanotube incorporation. The methods were compared using composites of five different PP types, each filled with 2 wt % MWCNTs. After the determination of the specific mechanical energy (SME) input in the MB dilution process, the direct-incorporation mixing time was adjusted to achieve comparable SME values. Interestingly, the electrical resistivity of MB-prepared samples with 2 wt % MWCNTs was higher than that of those prepared using direct incorporation—despite their better dispersion—suggesting more pronounced MWCNT shortening in the two-step procedure. In summary, this study on PP suggests that the masterbatch approach is suitable for the dispersion of MWCNTs and holds advantages in nanotube dispersion, albeit at the cost of slightly increased electrical resistivity.

## 1. Introduction

The quality of the dispersion of carbon nanotubes (CNTs) in melt-mixed composites is one of the main influencing factors on electrical and mechanical properties [1,2,3,4,5,6,7,8,9,10]. The potential of nanotubes can be only fully exploited when the as-produced primary CNT agglomerates are individualized on the nanoscale and suitably distributed in the matrix. If primary agglomerates remain undispersed, higher loadings are required to achieve electrical percolation and, thereby, the sought improvement in electrical properties. Agglomerates also may cause problems in shaping processes and act as stress concentrators, inducing earlier break under mechanical load. The achieved state of dispersion depends on properties of the nanotube material and the processing way and conditions [6,10,11]. Considering the nanotubes, their geometrical properties (length, diameter, waviness), structural properties (defect density), surface properties (functional groups, impurities) and their agglomeration state in the as-produced primary agglomerates are important. On the side of the polymer, its molecular weight (influencing the melt viscosity), surface properties and functional or reactive groups are of interest. Within the melt mixing processes, the CNT incorporation strategy, shear and elongation rates/stresses, mixing time, and temperature (influencing polymer viscosity and shear conditions) are some of the influencing parameters [10]. Additionally, the cooling conditions of the prepared composites to solid samples during the shaping step may influence the state of dispersion, especially for partially crystalline polymers. Secondary agglomeration of individualized nanotubes in the polymer melt during annealing and/or shear is another effect influencing the state of dispersion which was shown to be favorable for the formation of electrical CNT networks, needed for electrical conductivity [12,13,14,15]. However, electrical conductivity at a given CNT loading is not only dependent on the state of dispersion, distribution and network formation but also on many other factors, like intrinsic conductivity of matrix and CNTs, CNT length, interactions/reactions at the polymer-CNT interface, orientation, and alignment of CNTs [16].

Melt-mixed CNT composites may be produced using different approaches. On the one hand, direct incorporation (DI) of CNTs is used in many scientific studies and the influences of processing conditions on dispersion and electrical and other properties have been well-documented. In this approach, polymer and CNTs are either added to the mixing equipment in a premixed state or CNTs are added to molten polymer. Concerning the mixing parameters, it was shown that the different conditions and parameters can be summarized in the specific mechanical energy (SME) input and that SME significantly influences the state of dispersion. In general, the macro dispersion of CNTs is improved with an increase in SME input [7,17,18,19,20,21]. The findings on the influence of SME input on electrical resistivity are inconsistent [17,18,20], as more influencing factors than only dispersion contribute to it. It has been shown that CNTs are shortened during melt processing, and that CNT length decreases with SME input, which has deleterious consequences on the electrical resistivity [19,20]. On the other hand, shorter CNTs are easier to disperse. 

On the other hand, the masterbatch (MB) dilution approach is quite common, especially in industrial applications. Here, a masterbatch with CNT loadings of between 10 and 25 wt % guarantees that the nanotubes are bound by the polymer, greatly easing their handling. In contrast to DI, no direct handling of the CNTs is required, which eliminates the need for appropriate safety precautions. In the dilution process, the masterbatch matrix should be miscible with the diluting polymer and the already-existing nanotube network structure should be expanded by incorporation of polymer chains from the diluting polymer [10]. Different to the DI approach, the polymer is molten twice. Given the pre-existence of a CNT network, the two-step MB procedure is expected to yield improved nanotube dispersion compared to that achievable using DI. Unfortunately, information regarding the exact grade of polymer and type of nanotubes used in industrially produced masterbatches is not always available. This can sometimes be a problem as the polymer source in the masterbatch is typically chosen to have a much lower melt viscosity than typical commercial polymers to allow dispersion of high filler loadings. Thus, good dispersion was not always achieved, as illustrated in a study of Mičušík et al. [22], where MB islands in different polypropylene matrices were found. Besco et al. [23] diluted a polypropylene/multiwalled carbon nanotubes (PP/MWCNT) masterbatch with PP types of different viscosities. The lowest number of residual agglomerates and the lowest percolation threshold were found using a low viscosity PP which corresponds to the study of Mičušík et al. [22]. This shows that when PP with low matrix viscosity is used, the infiltration of the polymer chains into the already existing CNT network is more pronounced. In a study on PP/MWCNT composites comparing composites prepared using the DI and MB approaches, better dispersion and lower electrical resistivity were observed for twin-screw extruded and compression-molded MB samples [24]. However, SME was not considered in that study, so a direct comparison is not appropriate.

With regard to the electrical properties of composites, not only the state of the nanotube dispersion but also the nanotube shortening effect during melt mixing must be taken into account. The shorter the CNTs, the higher the CNT content required to form the electrical conductive network in the composite. Several authors have dealt with CNT shortening during processing. The shortening of nanotubes occurs at structural defect sites such as nanotube kinks (e.g., due to pentagonal and heptagonal carbon bonds present in entangled CNTs or polar groups on nanotube surfaces such as C–O, C=O and O–C=O) due to the mechanical stress acting on the CNTs during processing. For dry or wet ball grinding, a significant CNT shortening with increasing milling time was observed [25,26,27,28,29,30]. For CNT dispersion using ultrasonic, Chen et al. [31] described that the CNT length decreases with increasing ultrasonic energy and at the same time the width of the length distribution is reduced. For aqueous MWCNT dispersions, Fuge et al. [32] found a linear decrease of the mean MWCNT length with increasing ultrasonication time. The reason for the shortening of the CNT length in the dispersion of the CNT with the multi-pass friction stirring process is primarily the large plastic strain during processing [33]. Additionally, for melt dispersion of CNTs, significant length reduction was found, as described by Krause et al. for multiwalled CNT [11,20,30,34,35,36,37,38]. The CNT reduction can be up to 50% of the initial length and becomes more pronounced with increasing energy input. This finding was reported for polycaprolactone (PCL)/MWCNT composites [20], polycarbonate (PC)/MWCNT composites [35,37,38], polystyrene (PS)/MWCNT composites [39], and PC/styrene-acrylonitrile (SAN)/MWCNT composites [37]. For PC/MWCNT composites the influence of matrix viscosity on the shortening was evaluated showing significantly shorter nanotubes dissolved from the composite with the higher matrix viscosity [35]. Furthermore, the CNT concentration in the composite influences the intensity of length reduction, as the comparison of PC-based composites with 0.5 and 1 wt % in [37] shows. Already at these low CNT loadings, the initial CNT aspect ratio of 134 reduced to 93 (0.5 wt %) or 75 (1 wt %). It can therefore be expected that the CNT shortening during the MB production step will be significantly more pronounced than in DI.

This study helps to determine whether dilution of MB is a more effective form of nanofiller dispersion than the direct incorporation of nanotubes. The types of polymer and CNT and the processing equipment were kept constant to exclude some of the influencing factors. Based on the assumption that SME can provide a suitable measure to compare both approaches, PP based composites with loadings of 0.5 to 7.5 wt % MWCNTs (Nanocyl^TM^ NC7000) were prepared by melt mixing using a DSM Xplore 15ccm co-rotating microcompounder. This device was chosen because it allows easy measurement and adjustment of the SME by varying different parameters, whereby we have chosen the variation of the mixing time. A comparison was made between the MB dilution and direct nanotube incorporation methods, based on the state of nanotube dispersion and the electrical resistivity of composites based on three PP types with different molecular weights. In addition, two maleic anhydride (MA) modified PPs were used. To allow the comparison of the two incorporation procedures, the SME input was recorded during MB processing in the microcompounder and held similar by adjusting the mixing time used during direct incorporation.

## 2. Materials and Methods

Commercially available MWCNTs (Nanocyl^TM^ NC7000 (Nanocyl S.A., Sambreville, Belgium)) with a carbon purity of 90%, an average diameter of 9.5 nm and an average length of 1.3 µm [34] were used. Two grades of MA-modified polypropylene (PP) with different melt viscosities, Orevac^®^ PPC and Orevac^®^ 18732, were selected. In addition, three grades of unmodified polypropylene were used: Moplen HP501H, a PP matrix material which is used for CNT-filled masterbatches from Hyperion Catalysis International Inc. (unknown trade name, marked as PPH), and Moplen HP400R. Relevant properties are given in Table 1.

A DSM Xplore microcompounder (Sittard, The Netherlands) with a filling volume of 15 cm^3^ and co-rotating twin-screws was used to prepare composites containing 1–6 wt % CNTs and the masterbatches (7.5 wt % CNTs). Polymer and MWCNTs were added simultaneously via the hopper into the running compounder. After compounding for 5 min at 250 rpm and 210 °C, composite strands were extruded. The masterbatch dilution step was performed under the same conditions. To measure the electrical resistivity, compression-molded plates (thickness 0.5 mm, diameter 60 mm) were prepared using a press PW40EH (Paul-Otto Weber GmbH, Remshalden, Germany) at 210 °C and 50 kN for 2 min.

Based on the force values recorded during the compounding, the specific mechanical energy SME was calculated using the following equation, as introduced by Krause et al. [17]

(1) $$\mathrm{SME}=\frac{2\pi \mathrm{Nr}}{m}\int_{t_{\mathrm{Start}}}^{t_{\mathrm{End}}}\mathrm{Fdt},$$

where N is the rotation speed of the microcompounder (250 rpm), r is the distance from rotation axis (0.002 m), t is the mixing time (5 min) and F is the force (in Newton), which is recorded. The SME input during direct incorporation is named SME_direct_. The SME applied to the composites produced by the masterbatch dilution approach (SME_MB_) was calculated by adding the calculated energy input in the masterbatch material mass used in the dilution to the calculated energy input during the diluting process:

(2) $${\mathrm{SME}}_{\mathrm{MB}}=\left(\frac{2\%}{7.5\%}{\mathrm{SME}}_{\mathrm{MBP}}\right)+{\mathrm{SME}}_{\mathrm{MBD}},$$

where SME_MBP_ is the SME applied in the masterbatch production (corresponds to SME_direct_ for 7.5 wt %) and SME_MBD_ the SME applied in the masterbatch dilution process from 7.5 wt % to 2 wt %. 

To make the comparison between MB dilution and DI at similar mechanical energy inputs (SMEs), an adapted mixing time t_adapted_ (applied in full minutes) was calculated using the following equations:

(3) $$\frac{{\mathrm{SME}}_{\mathrm{MB}}}{{\mathrm{SME}}_{\mathrm{direct}}}=\frac{t_{\mathrm{adapted}}}{t_{\mathrm{direct}}},$$

(4) $$t_{\mathrm{adapted}}=\frac{{\mathrm{SME}}_{\mathrm{MB}}*t_{\mathrm{direct}}}{{\mathrm{SME}}_{\mathrm{direct}}},$$

where t_direct_ is 5 min and the before calculated values of SME_MB_ and SME_direct_ were used.

**Figure 1:**
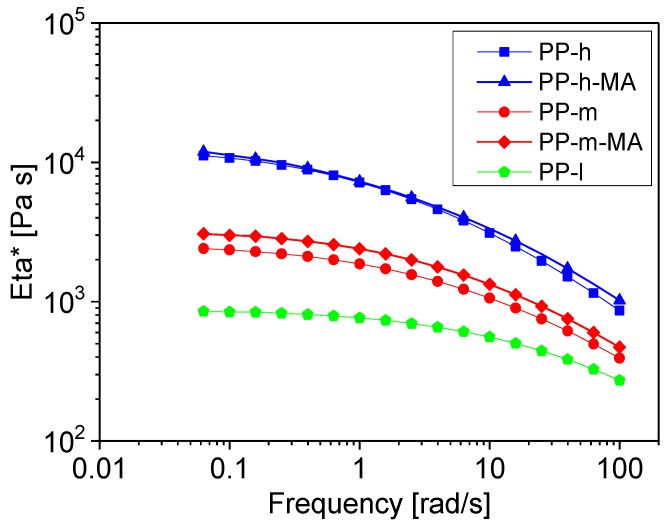
Complex viscosity |eta*| at 210 °C of the polypropylene materials.

Melt rheological measurements were performed on the neat PPs at 210 °C under nitrogen atmosphere, using an ARES oscillation rheometer (TA instruments, New Castle, DE, USA) with parallel plate geometry (diameter 25 mm, gap ca. 1 mm). Dynamic frequency sweeps (strain 10%) with increasing and decreasing frequency (between 0.05 and 100 rad s^−1^) were used, with the latter sweep used for interpretation. Figure 1 shows the obtained viscosity curves, illustrating the differences in viscosity and the comparability of PP-m and PP-h without and with MA. 

The state of MWCNT macro dispersion was studied using transmission light microscopy (LM) (Olympus BH2 with a DP71 camera, from Olympus Deutschland GmbH, Hamburg, Germany) on thin sections of 10 µm thickness. These were prepared from granules with 2 wt % MWCNT loading, using a Leica RM2155 microtome (Leica Microsystems GmbH, Wetzlar, Germany) equipped with a hard metal knife at room temperature. The agglomerate area ratio, A_A_, was calculated from LM images using six cuts, with a total area of ca. 3.2 mm^2^, of various granules using the digital image processing software ImageJ Version 1.43g (Bethesda, MD, USA). The area of agglomerates, A, was divided by the total area of the image A_0_. Only agglomerate areas with circle equivalent diameters larger than 1 µm were considered.

Electrical volume resistivities greater than 10^7^ Ohm·cm were measured on circular plates (thickness 0.5 mm, diameter 60 mm) using a Keithley 8009 Resistivity Test Fixture (Cleveland, OH, US), based on ring electrodes, combined with a Keithley Electrometer 6517A (open symbols—Figure 3, Cleveland, OH, USA). For resistivities less than 10^7^ Ohm·cm but greater than 10^3^ Ohm·cm, the Keithley Electrometer 6517A (Cleveland, OH, USA) was used in combination with a 4-point test fixture (gold contact wires with a distance of 16 mm between the source and 10 mm between the measuring electrodes, filled symbols—Figure 3). Therefore, strips (5 × 25 × 0.5 mm^3^) were cut from the plates. Values lower than 10^3^ Ohm·cm were measured using the 4-point test fixture in combination with the multimeter DMM2000 (filled symbols—Figure 3, Cleveland, OH, USA).

## 3. Results

**Figure 2:**
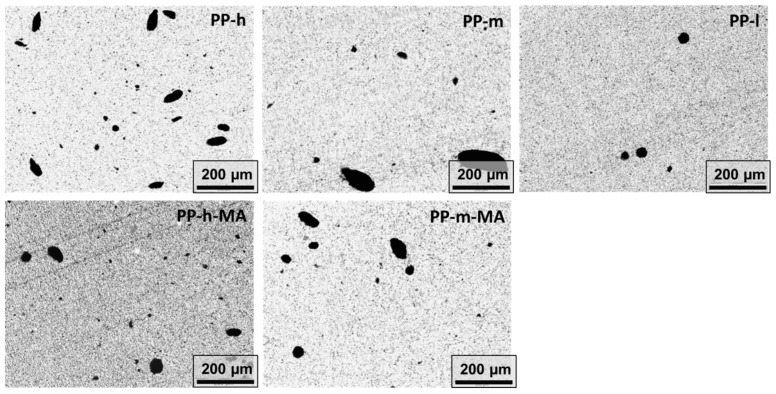
State of macro dispersion as observed by light microscopy for polypropylene (PP)/2 wt % multiwalled carbon nanotubes (MWCNT) composites.

In a first step, percolation sets were performed using the direct mixing approach with a constant mixing time of 5 min for all PP materials. Based on LM investigations on PP composites filled with 2 wt % MWCNT (Figure 2, Table 2), it was found that dispersion improves with decreasing matrix viscosity. In a study on polycarbonate by Kasaliwal et al. [40], improved dispersion was reported with an increase in matrix viscosity when using constant mixing conditions, but comparable levels of applied stress yielded reduced dispersion. This indicates the importance of melt infiltration into the primary agglomerates as first step of the dispersion process, which is more pronounced at lower matrix viscosity. 

MA-modified matrices were found to allow improved CNT dispersion (lower agglomerate area ratio A_A_) compared to unmodified matrices at similar melt viscosities, indicating the compatibilizing effect of the anhydride groups. This finding is in good agreement with Pan et al. [41] who compared PP and PP-*g*-MA composites filled with 1 wt % MWCNT.

**Figure 3:**
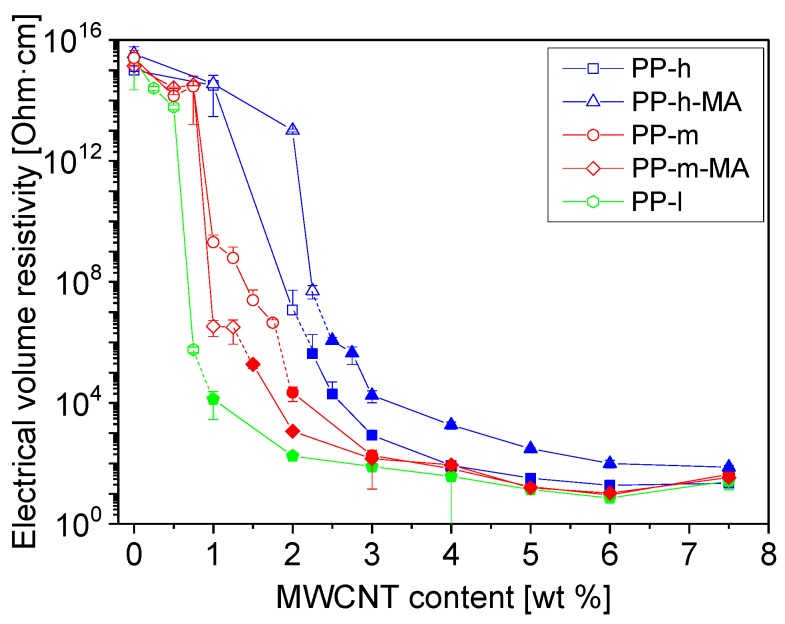
Electrical volume resistivity of the composites prepared by direct incorporation (5 min) of MWCNTs.

The electrical properties of the composites depend on matrix viscosity and MA modification, as illustrated in Figure 3. For the unmodified PPs, the electrical nanotube percolation threshold is lower at lower matrix viscosity. It is about 0.75 wt % for composites based on PP-l, ca. 1 wt % for PP-m, and 2 wt % for PP-h based composites. This dependence is in accordance with previous reports on PP and other polymer matrices [23,35,40,41]. The higher percolation threshold at higher matrix viscosity seems to result from the combination of decreased dispersion combined with (expected) more intensive nanotube shortening. In addition, the lower matrix viscosity promotes secondary agglomeration during the compression molding step, also contributing to lower electrical resistivity. Interestingly, a resistivity plateau develops from 4 wt % loading and the differences between the composites having different matrix viscosity disappear. MA-grafting increases the CNT percolation threshold for PP with high viscosity which corresponds to the findings of Pan et al. [41]. Next to the compatibilizing effect of the MA-groups, a wrapping of the nanotubes by grafted polymer chains can also be expected which leads to the formation of insulating layers around nanotubes hindering the conductive network formation [41]. In contrast, the electrical percolation threshold is reduced in the case of medium viscosity PP when MA-grafted PP is used indicating the in this case the compatibilization effect is dominant.

In the second set, composites with 2 wt % MWCNT loadings were produced using direct incorporation at 5 min and masterbatch dilution as mentioned above. In all cases, the two-step masterbatch dilution method resulted in significantly better macro dispersion (shown as values of A_A_ in Table 2) independent of the PP viscosity and MA modification. However, when calculating the applied SME values, higher mechanical energies were applied in the MB approach, indicating that the results cannot be compared directly. Thus, comparable SMEs were sought through an increase in the direct incorporation mixing time (see Table 3). Even if this aim could not be completely achieved (slight differences in SME persist), some general tendencies can be observed. In all cases, the MWCNT dispersion is improved when using the MB-dilution approach (Table 2 and Table 3) indicated by lower values of agglomerate area ratio. 

**Figure 4:**
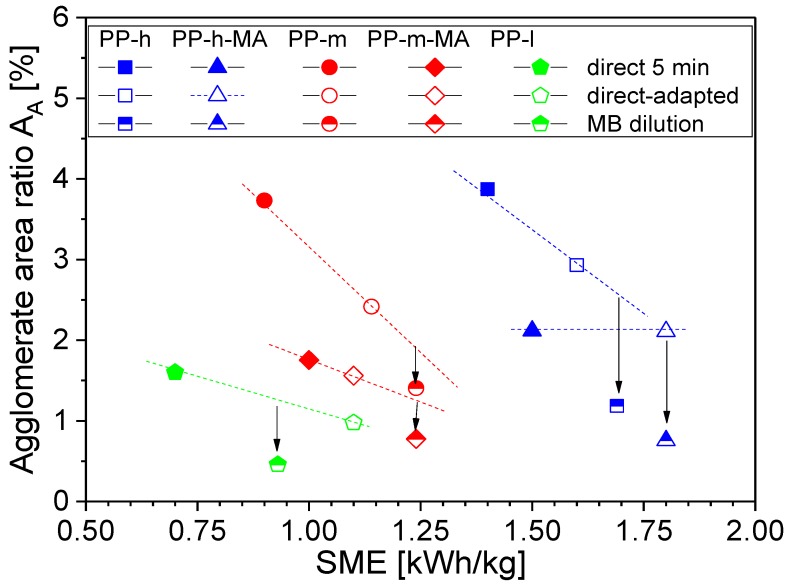
State of macro dispersion (A_A_) vs. SME for PP/2 wt % MWCNT composites prepared using different conditions, lines and arrows are only for guiding the eyes.

The dependence between the agglomerate area ratio and the SME is plotted in Figure 4. Regarding each polymer grade, A_A_ decreases with SME (except PP-h-MA). This finding corresponds to results on PC/MWCNT composites, in which mixing time and rotation speed in the same mixer were varied and dispersion vs. SME was plotted and formed a master curve [18]. However, the different PP types used here show different positions in the A_A_ vs. SME dependency in Figure 4 and no general trend can be observed across all mixtures. When plotting a trend line for each polymer, it is clearly visible that all composites prepared by the MB approach have values below this line. There are several reasons for this finding. The main could be that the time entry of the SME, which is here regarded as an integral, is different in MB and DI processes. Due to the much higher concentration of the MB (3.75 times higher than in DI) higher shear stresses act on the primary CNT agglomerates during its preparation helping in their dispersion but also contributing to more excessive CNT shortening. Such shortened CNTs can be dispersed more easily, which also contributes to a better state of dispersion. The higher composite viscosity at high CNT content leads to high local SME values, especially at the beginning of the dispersion process when the polymer matrix has not completely melted or is below the set mixing temperature. This high local SME intensity applies for the second time during the dilution step. The already achieved state of CNT dispersion from the masterbatch preparation step can be improved in the dilution step, whereby the same processes of polymer wetting and infiltration of polymer chains in remaining primary agglomerates, rupture, dispersion and distribution occur again. In addition, more pronounced polymer degradation cannot be excluded when melting the polymer twice, which could result in viscosity reduction, also contributing to better dispersion.

**Figure 5:**
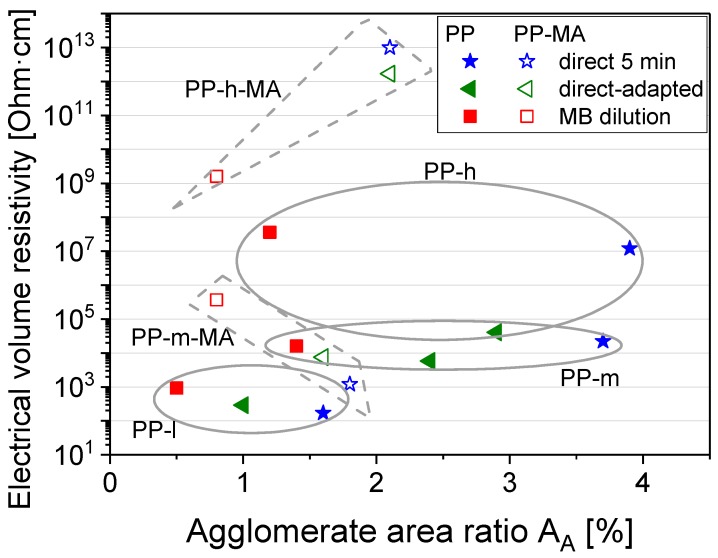
Electrical volume resistivity vs. state of macro dispersion (A_A_) for unmodified PP/2 wt % MWCNT composites, circles and polygons are only for guiding the eyes.

A direct correlation between electrical resistivity and macro dispersion cannot be found, as seen from Figure 5. Even if it is expected that an improved dispersion will lead to a lower electrical resistivity, it must be borne in mind that, as already mentioned, electrical conductivity depends on more factors. Next to the macro dispersion, these are for example nano dispersion and nano distribution, nanotube length, polymer wrapping, and matrix crystallinity. However, some trends can be seen in the grouping of data points. The composites prepared in different ways on the basis PP-l all have relatively low area ratios and the lowest resistivity values. Despite the lowest agglomerate area ratio, the MB diluted sample has the highest volume resistivity. For PP-m based composites there are larger differences in the A_A_ values, but the volume resistivities, which are all higher than for PP-l based materials, do not differ much. For PP-h, the A_A_ values differ the most across all PP matrices and the composite materials produced by DI with adapted mixing time have the lowest resistivity. Interestingly, the electrical resistivities of the MB dilution samples were similar (PP-m) or higher than those of the samples prepared with DI, despite the better dispersion (Figure 5). It should be noted that the MWCNT loading of 2 wt % used is above the corresponding percolation threshold for PP-l- and PP-m-based composites, but is at the percolation concentration for PP-h. This explains the generally higher resistivity values and the highest sensitivity to small structural changes. As shown for PC based composites, improving dispersion above a certain limit does not result in improved conductivity [18]. When comparing the resistivity values of PP-h based composites, the DI with adapted (longer) mixing time leads to the lowest resistivity. Such a tendency can also be observed for PP-m. The high resistivity values for all MB diluted samples indicate a stronger MWCNT length reduction in the two-stage MB process. As shown in an earlier study of PC-MWCNT composites with different CNT contents, shortening is more pronounced at higher CNT loadings [37]. The difference between the resistivities obtained after the MB and the DI approaches with comparable SME is greatest in PP-h, where the highest melt viscosity and highest shear stresses during compounding are expected to result in the most dramatic CNT length reduction. In the samples with PP-MA, two of the samples based on PP-h-MA were non-conductive, so these trends cannot be discussed. For PP-m-MA, the MB dilution showed the lowest A_A_ (Table 2, Figure 5) but the highest resistivity of all PP-m based samples. The values for PP-m-MA based composites were in the range of PP-m.

**Figure 6:**
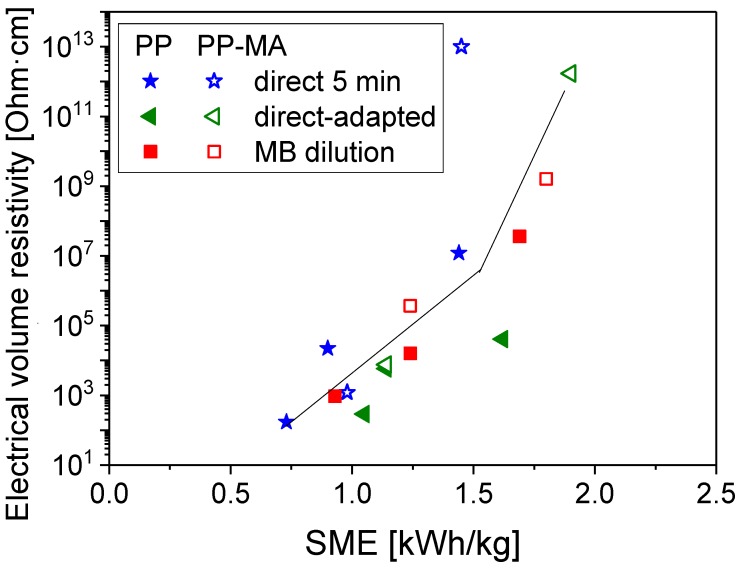
Electrical volume resistivity vs. SME for PP/2 wt % MWCNT, lines are only for guiding the eyes.

When plotting the dependence of the electrical volume resistivity to SME input for all composites with 2 wt % MWCNTs (Figure 6), the tendency of an increased resistivity can be observed at higher applied SME. This general trend is independent of the method used to prepare the samples and of the PP matrix viscosity used. Such trends have previously been described for polyamide 6 (PA6)/MWCNT [17] and PCL/MWCNT [20] based composites for SME values above 0.5 kWh/kg. In the present study, all samples prepared by DI with adapted mixing time have data points slightly below the trend line indicating the best conductivity at a given SME. The increase in resistivity with SME was not to be expected a priori, but is a clear indication that it is not only the improved dispersion that determines the electrical properties. We assume that a main influencing factor was the nanotube shortening, as shown in earlier studies [19,20,35,37,38]. 

## 4. Conclusions

Based on the example of PP/MWCNT composites, it could be shown that, when performed with similar mechanical energy input, the masterbatch dilution technique resulted in better MWCNT macro dispersion than that achievable by the direct incorporation. This finding was independent of the polypropylene matrix viscosity and the PP modification with MA. Despite better macro dispersion, the electrical resistivity of compression molded samples with 2 wt % MWCNTs was slightly higher in the case of low and medium viscosity PP, and significantly higher with high viscosity PP when using the masterbatch approach. Among the numerous factors influencing electrical resistance, it is assumed that nanotube shortening plays an important role in the development of conductive networks. An increased SME, which is expected to lead to a stronger shortening, contributes significantly to the observed increased resistance values.

MA modification of the PP matrices led to even higher electrical resistivity values, even though an improved dispersion compared to the corresponding unchanged PP matrices was found due to the compatibilization effect. This may be caused by a polymer wrapping effect on the nanotubes that interferes with the conducting network formation.

In summary, it can be stated that the masterbatch approach is very well suited for the dispersion of MWCNTs since it leads to a good CNT dispersion and only slightly increased electrical resistance values of the melt mixed composites compared to direct incorporation.

## Figures and Tables

**Table 1:** Properties of the five polymer matrices.

Trade Name	Notation	Manufacturer	MFI [g/10 min]	MA-Content [wt %]
Moplen HP501H	PP-h	LyondellBasell (Rotterdam, The Netherlands)	2.1	-
Orevac^®^ PPC	PP-h-MA	Arkema Group (Colombes, France)	2.0	0.14 [22]
PPH	PP-m	-	11.8	-
Orevac^®^ 18732	PP-m-MA	Arkema Group (Colombes, France)	6–10	0.13 [22]
Moplen HP400R	PP-l	LyondellBasell (Rotterdam, The Netherlands)	25	-

**Table 2:** State of dispersion (agglomerate area ratio A_A_) for composites with 2 wt % MWCNT prepared using direct incorporation and masterbatch dilution (masterbatch with 7.5 wt %, diluted to 2 wt %); preparation at 210 °C, 5 min, 250 rpm.

Material	Direct Incorporation A_A_ [%]	Direct Incorporation SME [kWh/kg]	Masterbatch Dilution A_A_ [%]	Masterbatch Dilution SME [kWh/kg]
PP-h	3.9 ± 1.4%	1.44	1.2 ± 0.7%	1.69
PP-h-MA	2.1 ± 0.9%	1.45	0.8 ± 0.2%	1.80
PP-m	3.7 ± 2.2%	0.90	1.4 ± 1.0%	1.24
PP-m-MA	1.8 ± 0.7%	0.98	0.8 ± 0.2%	1.24
PP-l	1.6 ± 0.7%	0.73	0.5 ± 0.1%	0.93

**Table 3:** Calculated adapted mixing time, state of dispersion (agglomerate area ratio A_A_) for composites with 2 wt % MWCNT prepared using direct incorporation (210 °C, adapted time, 250 rpm) and real specific mechanical energy (SME) measured under adapted conditions.

Material	Adapted Mixing Time [min]	Direct Incorporation Adapted A_A_ [%]	Direct Incorporation Adapted SME [kWh/kg]
PP-h	6	2.9 ± 0.9%	1.62
PP-h-MA	7	2.1 ± 0.7%	1.90
PP-m	7	2.4 ± 1.0%	1.14
PP-m-MA	6	1.6 ± 0.3%	1.14
PP-l	8	1.0 ± 0.4%	1.05
